# A Comparison of Endovascular Aneurysm Repair and Open Repair for Ruptured Aortic Abdominal Aneurysms

**DOI:** 10.7759/cureus.25672

**Published:** 2022-06-05

**Authors:** Samaher A Alnefaie, Yasser A Alzahrani, Bashair S Alzahrani

**Affiliations:** 1 Radiology, King Fahad Armed Forces Hospital, Jeddah, SAU

**Keywords:** ruptured endovascular aneurysm repair, abdominal aortic aneurism, rcts, aorta, open repair, evar, endovascular

## Abstract

Management modalities of ruptured abdominal aortic aneurysm (AAA) include ruptured open aneurysm repair (rOAR) and ruptured endovascular aneurysm repair (rEVAR). In this study, we aim to systematically review all the previously published randomized controlled trials (RCTs) that compared rOAR and rEVAR. A systematic search was performed in the following databases: PubMed, Scopus, Web of Science, Google Scholar, Clinical trials, and others with all the potentially relevant keywords that were adjusted to meet the search strategy for each database to collect all the relevant studies that were published up to January 2021. A total of 11 studies were identified through our comprehensive search. Among these studies, seven represented the IMPROVE trial, two represented the AJAX trial, and two represented the Nottingham and ECAR trials, each, while the remaining four studies were not RCTs; however, these were included in the discussion as they obtained data from the IMPROVE trial. The IMPROVE trials preferred EVAR use due to the potential survival benefit and improved quality of life, although the EVAR and OAR had similar mortality rates. In the AJAX and ECAR, the mortality rates favored EVAR over OAR with no significance while the opposite was noticed in the Nottingham trial with no significance also. Similar rates of re-interventions and complications were also noticed and some studies reported that EVAR is cost-effective. Overall evidence slightly favors EVAR over OAR and further studies are needed.

## Introduction and background

Abdominal aortic aneurysm (AAA) has a high prevalence rate of 4%-8% globally, and the risk increases over the age of 60 years old [1]. Reports from the United Kingdom, however, showed that the prevalence rate is decreasing which is suggestive of reduced cardiovascular system (CVS) risks and increased management over the past years [2,3]. Although the recent medical advances, management of AAA is a risky procedure that has many considerations and causes serious complications that may lead to death. This is also attributable to the event as most patients with AAA usually present with AAA rupture. Previous estimates show that AAA rupture is responsible for 53%-65% of in-hospital mortality events and for around 43% of most-interventional mortality events as obtained from investigations in developed countries [4]. Consequently, the AAA has been classified as the 15th cause of death in the United States population as reported by the Centers for Disease Control [5].

Management modalities of ruptured AAA include ruptured open aneurysm repair (rOAR) and ruptured endovascular aneurysm repair (rEVAR). Many complications have been associated with the rOAR procedure. Such complications include severe hemorrhage, surgical exposure and septicemia, and multiple organ failure. As a result of surgical-related clamping of the aorta, perfusion-related events can cause ischemia and cellular injury which is responsible for most of the subsequent complications. This can cause up to 30%-65% intraoperative death rates following this procedure [4,6]. On the other hand, rEVAR is a non-open surgical modality that was first introduced in 1994 and has gained remarkable popularity due to its advantages over the rOAR procedure (being less invasive, has more complications, and does not always require general anesthesia) [7-9]. However, performing rEVAR is not flexible and depends on the condition of each patient. Therefore, rEVAR can be considered optional in high-risk operations while rOAR remains the first choice [4,10,11].

Based on the previous information, no evidence can be found to favor one of the modalities over the other [12]. Various studies have compared the two modalities in terms of efficacy, complications, and cost-effectiveness to decide which modality was superior to the other. However, such outcomes were not assessed by many randomized controlled trials (RCTs). Previous systematic reviews have compared both modalities, however, they were either non-comprehensive or old and did not analyze the data from the available RCTs only which may be a cause for heterogeneity [13,14]. Thus, we aim to systematically review all RCTs that compared rOAR and rEVAR in all aspects that can be found within studies from the current literature.

## Review

Methods

Search Strategy

We have included all of the relevant keywords in our search strategy to obtain all the relevant studies that meet our inclusion criteria. Our systematic search was done through the following databases: PubMed, Scopus, Web of Science, Google Scholar, Clinical trials, the International Standard Randomized Controlled Trial Number, and the Cochrane Central Register of Controlled Trials. Our search results were defined to obtain potential RCTs that are published up to January 2021. Our used search term includes the following keywords: ([endovascular OR EVAR] AND [“ruptured abdominal aortic aneurysm” OR rAAA] AND [stent OR endograft OR “stent-graft” OR “emergency repair” OR open OR surgical OR OA OR OAR]). These keywords were adjusted per each database to obtain all the relative articles according to the guidelines of the search strategy of each database. Besides, we have manually searched the references of the included studies and the relevant systematic reviews to obtain any study that we could have missed by our search strategy.

Inclusion and Exclusion Criteria

After performing our comprehensive search strategy, we designed a detailed list of inclusion and exclusion criteria to ease the screening process and obtain all the possible articles according to our intended aims from the current study. For a study to be included, it should be: 1) an RCT, 2) compare between EVAR and OAR efficacies in rAAA patients, 3) have been published, and 4) have investigated human subjects only. On the other hand, we excluded the screened articles if they were: 1) non-RCT studies as observational and retrospective studies that did not perform any randomization, 2) RCTs that investigated the same outcomes among asymptomatic or unruptured AAA, 3) non-English articles, 4) thesis, abstracts, editorials, or protocols, or 5) animal studies.

Screening of the Imported Results

After the search process was complete, all of the obtained results were imported to an Endnote program library to identify and exclude all the possible duplicates between the different databases where the search strategy took place. After excluding the duplicates, the rest of the results were then exported to an Excel-specific sheet that is designed to make the screening process easy. After that, two independent reviewers took part in the screening process to find the relative studies based on our criteria. Whenever either of the reviewers was in doubt, this conflict was solved by a group discussion by the study participants and under the supervision of the corresponding author. The screening strategy included a title and abstract screening, followed by full-text screening to make sure the process is fast and effective to achieve the best results. These steps were done in accordance with the guidelines by the Preferred Reporting Items for Systematic Reviews and Meta-Analyses: The PRISMA Statement [15].

Data Extraction and Quality Assessment of the Included Studies

After the two reviewers have finished screening and made a list of the included articles, a pilot standardized excel sheet was then constructed by an experienced member who looked up some included articles and designed the sheet accordingly. The sheet was designed to collect all of the relative information from each study as the basic and characteristics, the outcomes of each article, and the elements of quality assessment of these articles. All of the study participants shared in this step and each conflict was resolved by a public discussion. The Cochrane Collaboration’s proposal for the assessment of the risk of bias (RoB 2) for RCTs [16] was the tool that we used in the present study. Assessment of bias was mainly dependent on the following domains: detection, selection, reporting, performance, and attrition. Each item was marked as low, unclear, or high according to the degree of bias in the assessed item and then each article was marked with similar domains based on the overall assessment scores.

Results

Search Results

**Figure 1:**
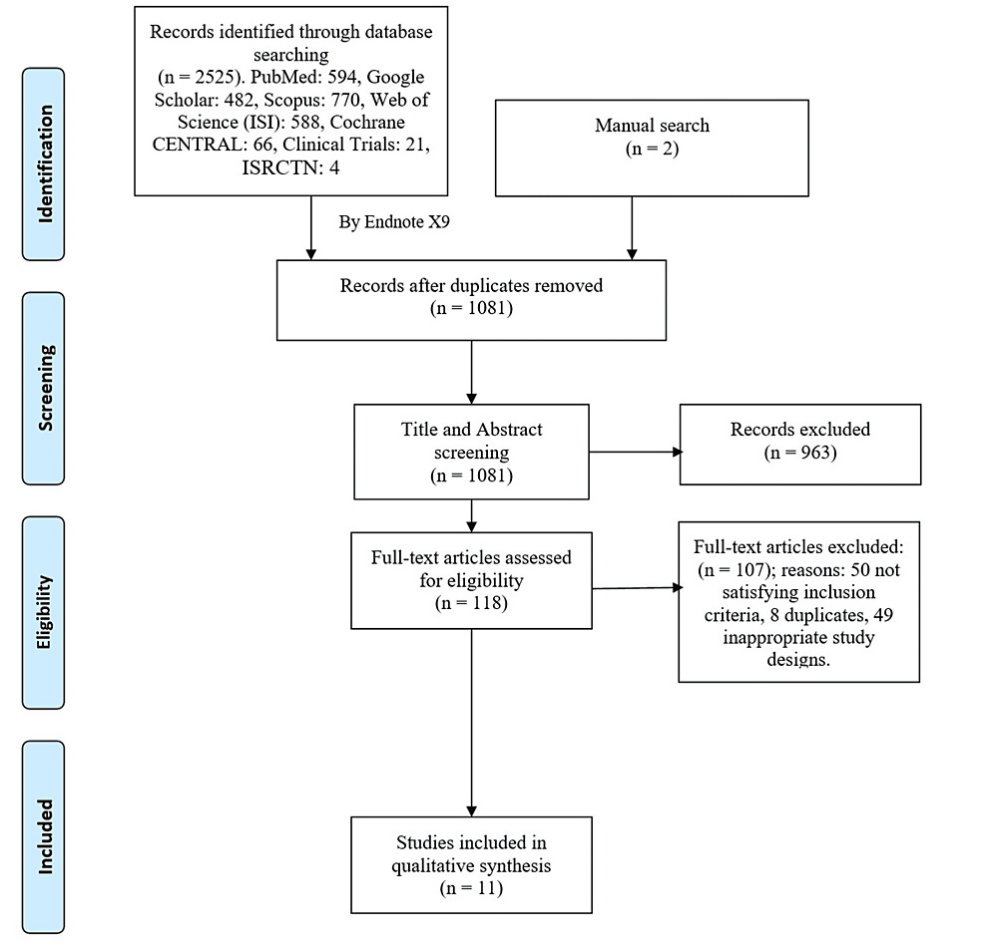
PRISMA flow chart for the search strategy and citations screening

After our comprehensive search in the aforementioned databases, all results were imported into an endnote library to remove all the duplicate citations among the different databases. Moreover, by manually searching the references of the included studies and previous systematic reviews, we managed to add additional two relevant articles. We found a total of 2,525 relevant articles that were shortened into 1,081 after duplicate removal. After title and abstract screening, a total of 963 citations were excluded, being non-RCTs, did not investigate the previously mentioned outcomes, and not written in English. Full-text screening was then performed and resulted in the inclusion of 11 relevant articles. A detailed information about the search strategy and data selection is presented in Figure 1.

Risk of Bias

**Figure 2:**
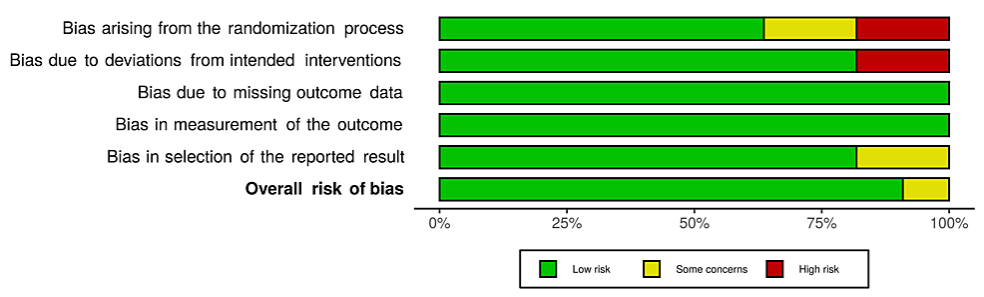
Risk of bias graph for the included randomized controlled trials

**Figure 3:**
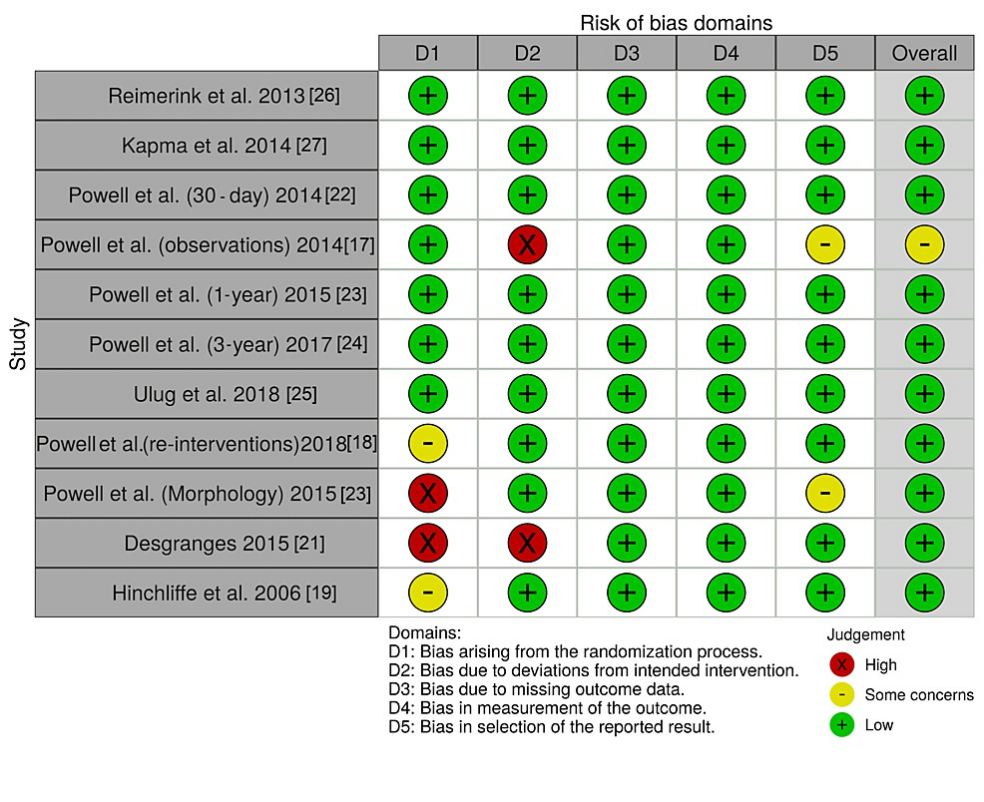
Summary for the included randomized controlled trials

Assessment of risk bias showed that almost all studies had a low risk of bias while only one [17] had some concerns. Assessment by domain showed that most studies had a low risk of bias in randomization of their population, while two studies [18,19] had some concerns and another two [20,21] had high risks of bias. The highest domain which may have had the highest degree of bias among the included studies is the randomization domain, while the lowest risk of bias was found in the measurement of outcome and missing outcome domains (Figure 2). Detailed information of all the assessed domains and the overall risk of bias for each study are presented in Figure 3.

Study Characteristics

The final list of our studies included eleven studies representing data from four RCTs. These include the Immediate Management of the Patient with Rupture: Open Versus Endovascular Repair (IMPROVE) trial [17,18,20,22-25], which is represented by six studies, the Amsterdam Acute Aneurysm (AJAX) trial which is represented by two studies [26,27], the Endovasculaire ou Chirurgie dans les Anévrysmes aorto-iliaques Rompus (ECAR) trial, which is represented by one study [21], and the Nottingham trial, which is represented by one study also [19]. We have also obtained four observational studies [28-31] that investigated the same outcomes using the same data from our included RCTs, however, these were excluded from our final list of the literature according to our criteria and were mentioned in the discussion section to make full use of the available data from the published RCTs. Most of these studies were published in 2014 [17,22,27] and 2015 [20,21,23] while two studies were published in 2018 [18,25], and three studies were published in 2006 [19], 2017 [24], and 2013 [26], each. Besides, the AJAX trial recruited patients from the Netherlands, the IMPROVE and Nottingham trials from the United Kingdom, while the ECAR trial from France. Other information regarding the sample size, intended outcomes, and net conclusions are presented in Table 1.

**Table 1:** Summary characteristics of the included studies and the net conclusion of their results CT: computed tomography, EVAR: endovascular aneurysm repair, OAR: open aneurysm repair, OR: odds ratio, RCT: randomized controlled trial, SD: standard deviation, QoL: quality of life

Reference	Trial name	Year	Study design	Sample size	EVAR	OAR	mean age (SD)	mean AAA diameter (SD)	Main outcome	Author conclusion
Reimerink et al. [26]	AJAX	2013	RCT	116	57	59	74.8	-	The composite of death and severe complications at 30 days	The primary endpoint rate was 47% for OR and 42% for EVAR with an absolute risk reduction of 5.4%; 95%CI: 13%-23%]. OR might be better than EVAR due to the unsuitability of some patients and the improved OR techniques.
Kapma et al. [27]		2014	RCT	116	57	59	-	-	30-day mortality, cost-effictiveness	EVAR is superior to OAR, however, is less affordable
Powell et al. [22] (30 day)	IMPROVE	2014	RCT	613	316	297	76.7 (7.6)	8.4 (1.9)	30-day mortality, 24 hour in-hospital mortality and cost-efficacy within 30 days	EVAR is not superior to OAR in the assessed outcomes.
Powell et al. [17] (observations)		2014	RCT	558	283	275	76.5	8.4	Effect of certain clinical factors on the efficacy of EVAR and OAR	Local anesthesia should be used with EVAR to improve the outcomes
Powell et al. [23] (1 year)		2015	RCT	613	316	297	76.7 (7.6)	8.4 (1.9)	1-year all-cause mortality, hospital stay, QoL	EVAR is not superior regarding the 1-year survival rate but is cost-effective, reduces hospital stay, and improves QoL
Powell et al. [24] (3 years)		2017	RCT	613	316	297	76.7 (7.6)	8.4 (1.9)	3-year all-cause mortality, hospital stay, QoL	EVAR is superior regarding the 3-year survival rate and is cost-effective, reduces hospital stay, and improves QoL
Ulug et al. [25]		2018	RCT	613	316	297	77	8.3	All of the previously reported outcomes from the previous IMPROVE trials were combined and analyzed in this study.	The trial showed that no significant differences were found at 30 days and one year, while at 3 years, EVAR was better in obtaining better survival rates and enhancing the corresponding patients' QoL due to the fast recovery.
Powell et al. (re-interventions) [18]		2018	RCT	502	259	243	76.2	8.5	The rate of re-interventions in the two groups between 0 and 90 days, and 3months and 3 years.	The rate of interventions was less among patients in the EVAR group but not statistically significant. Besides, amputations were more common in the OAR group.
Powell et al. [20] (Morphology)		2015	RCT	458	177	281	76	8.6	Relationship between aneurism morphology and outcomes in both groups	Patients' outcomes are affected by the aneurism morphology and not the approached modality
Desgranges et al. [21]	ECAR	2015	RCT	107	56	51	74.4	-	30-day and 1-year mortality, and cost-utility	EVAR and OAR are similar in all-cause mortality, however, EVAR is cost-effective
Hinchliffe et al. [19]	Nottingham	2006	RCT	32	15	17	75	85 (80-100)	30-day mortality rates, complications, and whether CT had a role in the outcomes	EVAR and OAR are similar in all-cause mortality and CT did not delay diagnosis and intervention

Discussion

All-Cause Mortality

The IMPROVE trial was the largest trial to compare between EVAR and OAR. The estimated 30-day mortality rates were 35.4% and 37.4% for the EVAR and OAR groups, respectively. However, no statistical significance was noticed between the two modalities [22]. In another study by the IMPROVE collaborators where the same data of the original IMPROVE was used to investigate the correlation between some clinical outcomes and their effect on the estimated 30-day mortality rate. The authors reported that both modalities had similar mortality rates, however, the rates in the EVAR group were significantly reduced after applying local anesthesia in these patients [17]. In another IMPROVE-based trial, 458 patients were randomized into 177 and 281 patients in the EVAR and OAR groups, respectively to study the effect of AAA morphology on patients’ outcomes. The reported 30-day mortality rates were 28.3% and 37.4% for the EVAR and OAR groups, respectively. Aneurysm neck length (per 16mm) was the only significantly correlated morphological factor [20]. The IMPROVE trial for assessment of the outcomes after one year of follow-up randomized 316 and 297 patients into the EVAR and OAR groups, respectively. The authors reported that no significant difference was found in the all-cause mortality rates between the two groups (EVAR= 41.1%, OAR= 45.1, p= 0.325) [23]. Another trial was also conducted by the IMPROVE collaborators to assess the outcomes after three years of follow-up. The authors found no significant differences were found between the total mortality rates in the EVAR and OAR groups whether at three months, more than three years, or all-follow-up periods. On the other hand, EVAR had significantly lower rates in the three months to three years period than the OAR group [24].

In the AJAX trial, the authors finally recruited 116 patients from three different centers and reported that patients with EVAR had a lower 30-day mortality rate (21%) than other patients in the OAR group (25%). Moreover, the six-month mortality rates were also in favor of the EVAR group which had a mortality rate of 28% which is lower than the OAR group (30.5%) [26]. It is worth mentioning that the same collaborators conducted an observational cohort study and the results were similar to the RCT indicating that none of the modalities can be deemed superior over the other [29]. In the Nottingham trial, which recruited 32 patients only, the authors reported that both the EVAR and OAR groups had similar 30-day mortality rates and complications, and therefore none of the modalities was deemed superior to the other [19]. It is worth mentioning that the authors recommended the application of EVAR for rAAA in a previous cohort study. However, the authors also reported that the overall mortality rate was 45% and in some patients, OAR was required to deal with the complications that result from EVAR [28]. In Kapma et al. [27], the authors reported that the total mortality rates after 30 days were 21% and 25% for EVAR and OAR, respectively, while at six months, the rates were 28% and 31%, respectively, with an estimated absolute risk reduction of 4.4 and 2.4 at both periods, respectively. No significant difference in 30-day mortality rates was found in Rollins et al. [31] populations within the two modalities. This was also supported by the results from the ECAR trial which showed that, although the EVAR group had lower mortality rates than the OAR one at 30 days and one year, such differences were not statistically significant [21]. 

Re-interventions

Rollins et al. [31] reported that similar re-intervention rates were estimated among patients within the two modalities. This cohort study was built on data from the IMPROVE RCT. None of the patients in the EVAR group from the Nottingham trial required reoperation while around 17.7% from the OAR group did [19]. In the AJAX trial, the reoperation rate was slightly higher in the EVAR group (24.6%) than the OAR one (20.3%), however, such differences were not statistically significant [26]. The IMPROVE trial collaborators also reported that both modalities had 30-day re-intervention rates [22]. In the AAA morphology IMPROVE trial, the authors reported that 13.8% and 26.5% 30-day re-interventions rates were done for the EVAR and OAR groups respectively. Besides, among the investigated AAA morphological aspects, none of them was significantly correlated with these rates [20]. The long-term results from the IMPROVE trial also indicated that the re-interventions rate was similar in both groups after one year [23]. Another trial was published by the IMPROVE collaborators to study the re-interventions rate after repairing the rAAA by either OAR or EVAR and for that the authors randomized 502 patients from the original IMPROVE trial that competed for a three-year follow-up after repair of rAAA. The study results showed that mid-term intervention rates were high for patients in the two modalities with no significant difference between both of them at three months and three years. Accordingly, better surveillance approaches should be made to intervene against such events and enhance the prognosis, especially in patients with OAR that are potentially prone to more complications [18].

Cost-Effectiveness and Quality of Life

Cost-effectiveness is an essential factor in the validation of any medical device or modality due to the potential impact on healthcare resources and quality of provided services. This outcome was assessed by seven studies that reported the following outcomes. In a study conducted by Rollins et al. [31], where the authors depended on data from the IMPROVE trial, they reported that no significant difference was found between the two modalities regarding the mean costs per patient and the mean total costs per life (p= 0.561). On the other hand, OAR had greater initial costs which were attributable to the prolonged hospital stay and procedure time while EVAR's long-term costs were higher due to the potential need for re-interventions and surveillance. This was supported by the ECAR trial as the results showed that OAR was associated with higher rates of complications which required longer hospital stays and consuming more resources [21]. On the other hand, the results from the Nottingham trial showed that patients in the OAR group had slightly more complications and longer hospital stays than patients in the EVAR one which raises questions about the previous claims by the ECAR trial about the cost-utility of the OAR modality [19]. Kapma et al. [27], on behalf of the AJAX trial also reported that EVAR modality was associated with higher costs, although it was associated with lower mortality rates. The authors reported that the total mean difference for costs in their population was 5,306 and 10,189 at 30 days and six months, respectively between EVAR and OAR. The authors justified the high costs for EVAR by arguing that eight patients that were indicated for the EVAR modality were then re-converted for performing OAR. Besides, no significant difference was noticed between the two groups regarding their QoL. Researchers from the IMPROVE trials also reported that the costs were similar for both modalities with an estimated 30-day cost difference of $1,939 for EVAR over OAR [22]. On a long-term basis, the IMPROVE collaborators concluded that EVAR was cost-effective, and patients had a better quality of life and faster discharge from hospitals [23]. No significant differences were also found between the two modalities regarding the quality of life, life years, and cost-effectiveness in the three-year IMPROVE trial [24].

In the recently published combined IMPROVE trial, the author combined the results and outcomes of all the previously published IMPROVE RCTs. No significant differences were noticed at 30 days and one year in terms of mortality; however, at three years, the results were in favor of EVAR due to a mid-term survival benefit. Moreover, EVAR was associated with less duration of hospital stays which improved the QoL for EVAR patients, being cost-effective. Therefore, the authors recommended EVAR over OAR for the management of rAAA [25]. We have also found three related systematic reviews during our screening. Badger et al. [14] analyzed the results of the AJAX, Nottingham, and IMPROVE at 30 days and reported that no significant differences were found between the two modalities in terms of mortality and other outcomes. In another systematic review, the author included 93 studies, including the four RCTs, and concluded that EVAR is not superior to OAR, although the authors did not perform any analysis and depended on some descriptive statistics based on data obtained from the included studies [13]. In a more recent meta-analysis, Kontopodis et al. [32] analyzed the results from 136 studies, including the four RCTs, and reported that both modalities showed favorable outcomes over the years; however, OAR operations overload was significantly associated with more risk of perioperative mortality which favors the use of EVAR. However, the significant difference obtained from the huge number of the observational studies might be attributable to the high risk of selection bias as suggested by Ambler et al. [30] that reported that a change in the survival benefit for EVAR over OAR was noticed after randomization of patients after the correlation was significant for EVAR. This suggests that further RCTs are still needed for further validation.

Limitations to our study include the limited number of the included trials which recommends the need to conduct further RCTs with a large sample size to help validate the current evidence. No pooled analysis was done in the present study and results were obtained from the relative RCTs and stated.

## Conclusions

The results from our study indicate that EVAR is better than OAR in obtaining favorable survival benefits. However, the results show that OAR is associated with similar rates of mortalities and complications, however, the inferiority of using OAR for EVAR results from the overburdened healthcare systems and patients by the increased hospital stays, potential re-interventions, and the subsequent cost-utility. Although many observational studies have recently been published, we recommended that further studies with big sample sizes and better randomization should be performed to furtherly validate these results and overcome the potential risk of bias in these observational studies.

## References

[1] Ashton HA, Buxton MJ, Day NE (2002). The Multicentre Aneurysm Screening Study (MASS) into the effect of abdominal aortic aneurysm screening on mortality in men: a randomised controlled trial. Lancet.

[2] Conway AM, Malkawi AH, Hinchliffe RJ, Holt PJ, Murray S, Thompson MM, Loftus IM (2012). First-year results of a national abdominal aortic aneurysm screening programme in a single centre. Br J Surg.

[3] (2021). Abdominal aortic aneurysm screening. https://www.nhs.uk/conditions/abdominal-aortic-aneurysm-screening/.

[4] Karthikesalingam A, Holt PJ, Vidal-Diez A (2014). Mortality from ruptured abdominal aortic aneurysms: clinical lessons from a comparison of outcomes in England and the USA. Lancet.

[5] (2021). Deaths, Percent of Total Deaths, and Death Rates for the 15 Leading Causes of Death in 5-year Age Groups, by Race and Sex: United States, 1999-2015. https://www.cdc.gov/nchs/nvss/mortality/lcwk1.htm.

[6] Lindsay TF, Luo XP, Lehotay DC (1999). Ruptured abdominal aortic aneurysm, a "two-hit" ischemia/reperfusion injury: evidence from an analysis of oxidative products. J Vasc Surgery.

[7] Yusuf SW, Whitaker SC, Chuter TA (1994). Emergency endovascular repair of leaking aortic aneurysm. Lancet.

[8] Greenhalgh RM, Brown LC, Kwong GP (2004). Comparison of endovascular aneurysm repair with open repair in patients with abdominal aortic aneurysm (EVAR trial 1), 30-day operative mortality results: randomised controlled trial. Lancet.

[9] Prinssen M, Verhoeven EL, Buth J (2004). A randomized trial comparing conventional and endovascular repair of abdominal aortic aneurysms. N Engl J Med.

[10] Peppelenbosch N, Yilmaz N, van Marrewijk C, Buth J, Cuypers P, Duijm L, Tielbeek A (2003). Emergency treatment of acute symptomatic or ruptured abdominal aortic aneurysm. Outcome of a prospective intent-to-treat by EVAR protocol. Eur J Vasc Endovasc Surg.

[11] EVAR trial participants (2005). Endovascular aneurysm repair and outcome in patients unfit for open repair of abdominal aortic aneurysm (EVAR trial 2): randomised controlled trial. Lancet.

[12] Nedeau AE, Pomposelli FB, Hamdan AD (2012). Endovascular vs open repair for ruptured abdominal aortic aneurysm. J Vasc Surg.

[13] Amato B, Fugetto F, Compagna R (2019). Endovascular repair versus open repair in the treatment of ruptured aortic aneurysms: a systematic review. Minerva Chir.

[14] Badger SA, Harkin DW, Blair PH, Ellis PK, Kee F, Forster R (2016). Endovascular repair or open repair for ruptured abdominal aortic aneurysm: a Cochrane systematic review. BMJ Open.

[15] Liberati A, Altman DG, Tetzlaff J (2009). The PRISMA statement for reporting systematic reviews and meta-analyses of studies that evaluate healthcare interventions: explanation and elaboration. BMJ.

[16] Higgins JP, Altman DG, Gøtzsche PC (2011). The Cochrane Collaboration's tool for assessing risk of bias in randomised trials. BMJ.

[17] Powell JT, Hinchliffe RJ, Thompson MM (2014). Observations from the IMPROVE trial concerning the clinical care of patients with ruptured abdominal aortic aneurysm. Br J Surg.

[18] Powell JT, Sweeting MJ, Ulug P, Thompson MM, Hinchliffe RJ (2018). Editor’s Choice - re-interventions after repair of ruptured abdominal aortic aneurysm: a report from the improve randomised trial. Eur J Vasc Endovasc Surg.

[19] Hinchliffe RJ, Bruijstens L, MacSweeney ST, Braithwaite BD (2006). A randomised trial of endovascular and open surgery for ruptured abdominal aortic aneurysm - results of a pilot study and lessons learned for future studies. Eur J Vasc Endovasc Surg.

[20] IMPROVE Trial Investigators (2015). The effect of aortic morphology on peri-operative mortality of ruptured abdominal aortic aneurysm. Eur Heart J.

[21] Desgranges P, Kobeiter H, Katsahian S (2015). Editor's Choice - ECAR (Endovasculaire ou Chirurgie dans les Anévrysmes aorto-iliaques Rompus): a French randomized controlled trial of endovascular versus open surgical repair of ruptured aorto-iliac aneurysms. Eur J Vasc Endovasc Surg.

[22] Powell JT, Sweeting MJ, Thompson MM (2014). Endovascular or open repair strategy for ruptured abdominal aortic aneurysm: 30 day outcomes from IMPROVE randomised trial. BMJ.

[23] IMPROVE Trial Investigators (2015). Endovascular strategy or open repair for ruptured abdominal aortic aneurysm: one-year outcomes from the IMPROVE randomized trial. Eur Heart J.

[24] IMPROVE Trial Investigators (2017). Comparative clinical effectiveness and cost effectiveness of endovascular strategy v open repair for ruptured abdominal aortic aneurysm: three year results of the IMPROVE randomised trial. BMJ.

[25] Ulug P, Hinchliffe RJ, Sweeting MJ (2018). Strategy of endovascular versus open repair for patients with clinical diagnosis of ruptured abdominal aortic aneurysm: the IMPROVE RCT. Health Technol Assess.

[26] Reimerink JJ, Hoornweg LL, Vahl AC (2013). Endovascular repair versus open repair of ruptured abdominal aortic aneurysms: a multicenter randomized controlled trial. Ann Surg.

[27] Kapma MR, Dijksman LM, Reimerink JJ (2014). Cost-effectiveness and cost-utility of endovascular versus open repair of ruptured abdominal aortic aneurysm in the Amsterdam Acute Aneurysm Trial. Br J Surg.

[28] Hinchliffe RJ, Yusuf SW, Macierewicz JA, MacSweeney ST, Wenham PW, Hopkinson BR (2001). Endovascular repair of ruptured abdominal aortic aneurysm--a challenge to open repair? Results of a single centre experience in 20 patients. Eur J Vasc Endovasc Surg.

[29] van Beek SC, Vahl A, Wisselink W, Reekers JA, Legemate DA, Balm R (2015). Midterm re-interventions and survival after endovascular versus open repair for ruptured abdominal aortic aneurysm. Eur J Vasc Endovasc Surg.

[30] Ambler GK, Twine CP, Shak J (2014). Survival following ruptured abdominal aortic aneurysm before and during the IMPROVE Trial: a single-centre series. Eur J Vasc Endovasc Surg.

[31] Rollins KE, Shak J, Ambler GK, Tang TY, Hayes PD, Boyle JR (2014). Mid-term cost-effectiveness analysis of open and endovascular repair for ruptured abdominal aortic aneurysm. Br J Surg.

[32] Kontopodis N, Galanakis N, Antoniou SA (2020). Meta-analysis and meta-regression analysis of outcomes of endovascular and open repair for ruptured abdominal aortic aneurysm. Eur J Vasc Endovasc Surg.

